# Epigenetic Drugs and Their Immune Modulating Potential in Cancers

**DOI:** 10.3390/biomedicines10020211

**Published:** 2022-01-19

**Authors:** Yingying Liang, Sevin Turcan

**Affiliations:** Neurology Clinic and National Center for Tumor Diseases, University Hospital Heidelberg, 69120 Heidelberg, Germany; yingying.liang@med.uni-heidelberg.de

**Keywords:** epigenetic drugs, antiviral response, epigenetics

## Abstract

Epigenetic drugs are used for the clinical treatment of hematologic malignancies; however, their therapeutic potential in solid tumors is still under investigation. Current evidence suggests that epigenetic drugs may lead to antitumor immunity by increasing antigen presentation and may enhance the therapeutic effect of immune checkpoint inhibitors. Here, we highlight their impact on the tumor epigenome and discuss the recent evidence that epigenetic agents may optimize the immune microenvironment and promote antiviral response.

## 1. Introduction

Epigenetic changes in cancer are diverse and dynamic and are emerging as potential targets for cancer therapies. Epigenetics is defined as reversible modifications that can lead to changes in gene expression without altering the DNA sequence [1]. The development of cancer as a chronic process is accompanied by dynamic epigenetic changes [2]. Cancer-associated epigenetic alterations may facilitate tumorigenesis or enhance acquired resistance to therapies [3]. Most genes that are aberrantly methylated are involved in pathways related to cell cycle, Wnt signaling, cellular invasion, and DNA repair [4]. Interestingly, the transition from stem cells to tumor cells often involves a group of tumor-initiating cells with epigenetic alterations, therefore, the possibility to reverse the potentially harmful epi-mutations can have significant therapeutic implications [5].

## 2. Epigenetic Modifications in Cancer

### 2.1. DNA Methylation

DNA methylation is the most common epigenetic modification in normal and cancer cells and typically involves the addition of a methyl group to a cytosine residue [6]. There are three major enzymes involved in DNA methylation: DNA methyltransferase 1 (DNMT1), DNMT2, and DNMT3s (DNMT3A/3B/3L). DNMT1 is the major enzyme involved in the stable inheritance of DNA methylation, while DNMT3A/3B play a predominant role in de novo DNA methylation. In normal cells, repetitive genomic regions such as long interspersed nuclear elements (LINE) and short interspersed nuclear elements (SINE) are typically hypermethylated [1]. LINE and SINE retrotransposons are the most common types of transposable elements (TE) [7]. In cancers, most of the aberrant DNA methylation is located within CpG islands. CpG methylation interferes with transcription factor binding or recruits methyl-CpG-binding domain (MBD) proteins and ultimately inhibits gene expression [1]. DNA hypomethylation is associated with tumor progression and occurs mainly in repeat DNA sequences in cancers [8]. For example, metastatic cancers have reduced levels of methylated cytosine compared to normal tissues. Wilms tumors and epithelial ovarian carcinomas are malignant lesions in which global DNA hypomethylation has been detected [9].

Aberrantly methylated genes can serve as biomarkers to predict therapeutic efficacy in cancers. A well-known example is the *MGMT* (O^6^-methylguanine–DNA methyltransferase) promoter methylation. Hegi et al. discovered that a subset of glioblastoma tumors with methylated *MGMT* responded better to the alkylating agent temozolomide (TMZ). The rationale was that the TMZ-mediated DNA mismatch is not corrected due to the hypermethylated and silenced *MGMT* gene [10]. The methylated DNA product of TMZ, termed as O^6^-methyguanine, can potentiate DNA single-strand break during mismatch repair, which in turn favors the p53-mediated apoptosis in *p53* wild-type tumors or facilitates the mitochondrial pathway of apoptosis in *p53* mutant tumors [11].

Promoter methylation of the mismatch repair gene *MLH1* correlates with microsatellite repeat instability in sporadic endometrial cancers [12]. Bisulfite sequencing of acute myeloid leukemia (AML) revealed that the extensive methylation of *MLH1* promoter might occur as a common molecular event in some AML patients [13]. Similarly, in microsatellite-instable colorectal cancers, the epigenetic silencing of *MLH1* together with concomitant gene repression in the chromosome 3p22 region likely contributes to the development of this tumor [14]. Acquired methylation of tumor suppressor genes may also be involved in tumor progression. For example, *CDKN2A* promoter hypermethylation increases when abnormal lesions progress from basal cell hyperplasia to carcinoma in situ in lung cancer [15]. While promoter methylation is associated with gene silencing, gene body methylation may facilitate gene transcription [16,17]. While intragenic and intergenic methylation may occur in the CpGi and non-CpGi regions, the distribution of methylated CpGi is more enriched in the gene body than in the 5′-promoters [18]. Methylation in the non-CpGi regions is a classic case for gene silencing of transposable elements (TEs) [17]. Local epigenetic alterations may be a specific feature of cancer. For example, promoter hypermethylation of p15 was exclusively found in leukemia while that of p16 only in colon cancers; in addition, aberrant methylation outside the promoters can distinguish the tumor-associated epigenetic changes [19]. Furthermore, DNA methylation patterns in tumor-initiating cells and senescent cells are distinct, suggesting that methylation changes inherent in cells which escape oncogene-induced senescence may contribute to tumorigenesis [20].

Indeed, both hypermethylation and hypomethylation could occur as a result of cancer-specific epigenetic modifications. Aberrant hypermethylated DNA occurs in CpG island-enriched promoters of tumor suppressor genes, whereas hypomethylation is usually global and hypomethylated repeat elements correlate with tumor progression. Sporadic DNA hypomethylation can be located in the gene body regions of oncogenes [21,22]. For example, *DNMT1*-depleted mice develop intestinal adenomas representing the onset of malignancy. The resulting DNA hypomethylation leads to genomic instability, which is considered characteristic of colorectal cancer [23]. Similarly, by comparing methylation levels at abundant CpG sites between hepatocellular carcinoma (HCC) and normal tissues, it was shown that the predominant cancer-associated epigenetic alteration is hypomethylation, which occurs mainly in intergenic regions, and contributes to genomic instability and tumorigenesis of HCC, while hypermethylation constitutes the rest of the cancer epigenome and is more likely to be found at promoters [24]. Interestingly, in the mouse model of hepatocarcinogenesis, significant hypermethylation was observed at CpG islands within the gene body and associated with overexpression of specific oncogenes such as *SCN8A*, *NFKB2*, *NEURL1B*, and *CDKN2B*. Notably, overexpression of these oncogenes also occurs in HCC patients, accompanied by hypermethylation of CpG islands within the gene body [25]. Hypermethylation at promoters of tumor suppressor genes such as *RASSF1A* and *APC* correlates with tumor grade of bladder cancer. The extent of hypermethylation in these regions may reflect the grade and invasiveness of bladder cancer [26]. In glioblastoma, recurrent hypomethylation within the gene body promoter was identified, including *TERT*, *GLI3*, and *TP73*, which may lead to oncogenic epigenetic and transcriptomic changes [27].

Epigenome-modifying genes are also altered in a number of tumors [28]. For example, recurrent isocitrate dehydrogenase (*IDH*) mutations are found in a number of cancers including myeloid malignancies and lower-grade gliomas (LGGs) [29,30] and establishes the CpG island methylator phenotype in glioma [31]. In prostate cancer, DNMT1 acts as tumor suppressor in the early stages of tumorigenesis, while contributing to metastasis as an oncogenic factor in the later stage [32]. In prostate cancer, a number of epigenetically silenced tumor suppressors have also been shown to be predictive of clinical features such as Gleason score and tumor stage [33].

DNA binding protein, the CCCTC-binding factor (CTCF), is a protein family that promotes or pauses DNA transcription and, together with other transcription factors, constitutes a complex network that determines specific transcriptional activity [34,35]. As a transcription regulator, CTCF represses *MYC*, by linking the promoter and enhancer domains [16]. Intergenic CTCF contributes to transcriptional repression by protecting local DNA hypermethylation. Interestingly, CTCF binding at exon 5 of CD45 was inhibited by DNA methylation, and this was inversely correlated with local 5-methylcytosine (5-mc) levels [36]. CTCF binding patterns in cancers may differ from those in normal tissues. Reduced CTCF binding is usually located within gene promoters while enhanced binding can be induced by oncogenic transcription factors and is related to enhancer regions [37]. Furthermore, CTCF deletion facilitates regional DNA hypermethylation in prostate and breast cancers and correlates with decreased expression of *TNFAIP3*, *FGF5*, and *EPHA3*. Inhibition of DNA methylation can facilitate re-expression of genes harboring CTCF binding sites [38].

The MBD (methyl CpG binding domain) proteins recognize DNA methylation sites and participate in transcriptional repression and heterochromatin formation [39]. They include MeCp2, MBD1, MBD2, MBD3, and MBD4. The distribution of MBD proteins and their affinity for methylated DNA depend on the gene promoter and cell type. For example, MeCp2 and MBD2 preferentially bind to promoters; however, no strong association of a particular MBD with specific promoters is indicated [40]. Following catalysis by relevant CpG methylation recognition enzymes, 5-mc can give rise to diverse modifications, including 5-hydroxymethylcytosine, 5-formylcytosine, and 5-carboxylcytosine, all of which together comprise 15 possible combinations. MBD1 and MeCp2 preferentially bind to 5mc/5mc or other 5mc-containing cytosine combinations [41].

### 2.2. Histone Modifications

Epigenetic deregulation of histones is also observed in cancer. In this review, we focus on the epigenetic regulation of histone methylation and histone acetylation. Histone acetylation mostly implies an active chromatin state (H3K9Ac, H3K14Ac, and H4K16Ac) and histone methylation marks can either be active and lead to gene activation (e.g., H3K4me2/3, H3K36me3, and H3K79me3) or repressive and lead to gene repression (H3K9me2/3, H3K27me3, and H4K20me3) [42].

Histone acetylation occurs by the addition of an acetyl group to H3 or H4, which interferes with the interaction between the core histones and DNA and facilitates transcription [11]. Histone methylation states have different functionalities. For example, H4K20me and H4K20me2 play a role in DNA replication and DNA damage repair, while H4K20me3 characterizes silenced heterochromatin [43]. H3K27me3 is mainly a mark for transcriptional repression, while H3K27me2 is involved in the control of enhancer activity. Co-expression of H3K27me1 and H3K36me3 is associated with open chromatin that promotes transcription [44].

Interestingly, G9a, a lysine methyltransferase that dimethylates H3K9, cooperates and colocalizes with DNMT1 during DNA replication. DNMT1 knockdown negatively affects G9a loading and H3 methylation [45]. Meanwhile, lysine methyltransferase SETDB1 is unable to convert its substrate to H3K9me3 in the presence of H3K4me3, and H3K9me3 catalyzing enzymes such as G9a, GLP, and SUV39H1 are unable to bind to DNA regions containing H3K4me3 [46]. Protein lysine methyltransferases and protein arginine methyltransferases are the two major classes of enzymes that catalyze histone methylation. In contrast, lysine demethylases remove the methyl-group from modified histones, which include KDM1A, KDM1B, and Jumonji C domain-containing demethylases [44]. The Enhancer of Zester homolog2 (*EZH2*), which contributes to the di- and trimethylation of H3K27 in mammals, is overexpressed in several tumors, including breast cancer, bladder cancer, and malignant melanoma. Moreover, *EZH2* overexpression is associated with poor survival in melanoma patients and its deletion results in slower tumor progression [47,48]. Similarly, low *EZH2* and H3K27me3 levels are predictors of better chemotherapy outcomes in ovarian cancer [49]. In addition, H3K27me3 has been shown to be an independent histone methylation marker for poor prognosis in bladder cancer [50] and has been associated with carcinogenesis and progression of prostate cancer [51]. H3K27me3 can function as a surrogate modification of hypomethylated TEs in taxane-resistant breast cancer cells, repressing their transcription and the resulting antiviral response. Breast cancer cells can be resensitized to taxane upon EZH2 inhibition [52]. On the other hand, loss of H3K27me3 in AML patients suggests shorter overall survival. Multivariate analysis revealed that reduced H3K27me3 in AML patients could serve as an independent unfavorable prognostic factor associated with an enhanced anti-apoptotic phenotype [53]. Loss of H3K27me3 has also been found to be an indicator of recurrence in meningiomas and a poor prognostic marker [54]. Concurrent high levels of H3K27ac and H3K27me3 were associated with aberrant *p53* and tumor aggressiveness in a subset of HCC [55]. The promoters of *PD-1*, *CTLA-4*, and *Tim3* were significantly hypomethylated in breast cancer compared to normal tissues, and increased expression of these genes and epi-modifications are likely to lead to tumor immune evasion. Additionally, the repressive histone markers H3K9me3 and H3K27me3 exhibited attenuated binding in these promoter regions [56]. H3K27 mutations lead to a global depletion of H3K27me3 and acquisition of H3K27ac, which is usually enriched in repeat elements, and the acquired H3K27ac increases the susceptibility of cells to epigenetic agents [57].

Furthermore, histone modifications, as essential components of the nucleosome, can affect DNA methylation and influence the accessibility of dinucleotides to DNA methyltransferases [17]. DNA methylation attracts MBD proteins, which in turn recruit the histone deacetylases (HDACs) and remove acetylation from histones, thereby repressing gene transcription [44].

## 3. The Cancer Epigenome Contributes to Antitumor Immunity

Epigenetic changes in immune cells may influence antitumor immunity. Inherent epigenetic events contribute to the regulation of several immune-related events, such as Ig expression, Th1 and Th2 differentiation, B cell maturation, cytokine expression, MHC I and II expression, and VDJ recombination [58]. Epigenetic changes in immune cells coordinate with pathogenic stimuli to alter immune cell plasticity. Epigenetic patterns distinguish innate and adaptive immune cells. Interferon-γ (IFN-γ) is essential for Th1 cells and these cells usually have a demethylated *IFN-γ* promoter; whereas memory CD8^+^ cells are characterized by having high H3K4me3 and low H3K27me3 at specific loci (*PRDM1*, *IFNG*, and *GZMB*) compared to naive T cells [59]. Naive CD4^+^ T cells often exhibit epigenetic suppression of both *IFN-γ* and *IL4*. The *IFN-γ* and *IL4* loci undergo DNA demethylation and histone acetylation when cells differentiate into Th1 and Th2, respectively. Notably, STAT4 and T-bet, STAT6 and GATA3 may serve as downstream signaling transducers in this process [60]. In another study, demethylation of H3K27me3 by demethylase JMJD3 was found to be a representative feature during CD4^+^ T cell activation through the JAK/STAT pathway [61].

Epigenetic agents have been shown to modify the anticancer immunity by enhancing the tumor-associated antigen presentation and recognition, as well as the effective function of cytotoxic T cells [62]. Compared to normal lymphocytes, increased DNA methylation has been found in chronic lymphocytic leukemia in association with higher DNMT1 levels. As a result, certain genes such as *E-Cadherin*, *p15*, and *p16* are silenced, whereas in normal cells these genes are demethylated and their expression is tightly regulated [63]. Aberrant methylation of the *CXCL14* promoter and subsequent gene repression have been observed in certain tumor types, including prostate cancer, gastric cancer, and colorectal cancer [64]. Meanwhile, Peng et al. found that the level of DNMT1 and EZH2 negatively affected the number of tumor infiltrating CD8^+^ lymphocytes and prognosis in an ovarian cancer model, and that epigenetic silencing of the chemokines *CXCL9* and *CXCL10* resulted in immunosuppression [65]. Epigenetic deregulation resulting from mutations of chromatin-modifying enzymes such as MLL2, EZH2, and EP300 is observed in follicular lymphoma and diffuse large B-cell lymphoma [66]. Overexpression of MeCP2 negatively affects Th1 cell differentiation in mice and leads to dysfunction of the cellular IFN-γ response by dampening the accessibility of the *IFN-γ* gene for transcription factor binding [67].

Bunsen et al. showed that autocrine 2-hydroxyglutarate (2-HG) produced by *IDH*-mutant gliomas inhibited the T cell activation and immunity [68]. Interestingly, de novo DNA methylation acquisition occurs during progressive T cell exhaustion and impedes T cell expansion and rejuvenation upon immune-checkpoint blockade (ICB). Furthermore, the exhaustion-related DNA modification was characteristic of tumor-infiltrating PD-1^hi^CD8^+^ T cells. DNA demethylating agents could reverse the exhaustion-associated changes, thereby bypassing effector T cell resistance to ICB, leading to improved tumor control [69]. Similarly, the upregulation of several immune checkpoints such as *Tim3*, *TIGIT*, and *PD-L1* was found in the peripheral blood of breast cancer and colorectal cancer patients in association with the corresponding promoter hypomethylation, while the DNA demethylating enzymes TET2 and TET3 also showed upregulation [70]. In summary, the epigenome is likely to play an integral part in shaping the immune cell landscape, immune evasion, and establishing tumor immunogenic signatures (“hot or cold” tumors) [71].

## 4. Targeting Cancer Epigenetics

### 4.1. Epigenetics: A Versatile Therapeutic Target

Several malignancies harbor mutations that lead to epigenetic alterations, such as the *IDH* mutation in gliomas [29,30]. *IDH* mutation leads to extensive DNA hypermethylation and blocks cellular differentiation [72]. The metabolite product of mutated *IDH*, 2-HG, is a competitive inhibitor of enzymes that utilize α-ketoglutarate, such as the Jumanji C domain containing histone demethylases JHDM1, lysine demethylase 4, and DNA demethylase TET2. DNMT inhibitors (DNMTi) alter the epigenetic landscape, which promotes cellular differentiation and suppresses cell growth [73]. Surprisingly, the attenuated DNMT1 achieved by DNMTi may not be sufficient to lead to global hypomethylation and re-express silenced genes. Therefore, epigenetic agents could be combined with alternate therapies to achieve the desired antitumor effect [74]. Due to the immunogenic nature of IDH1 (R132H), vaccine-based therapy targeting the mutation may be a potent therapeutic regimen [75]. In addition, DNMT1i azacytidine represses the growth of *IDH1* mutant gliomas in vivo, accompanied by hypomethylation and marked cellular differentiation, with no tumor recurrence observed up to 7 weeks after drug withdrawal [76].

**Figure 1:**
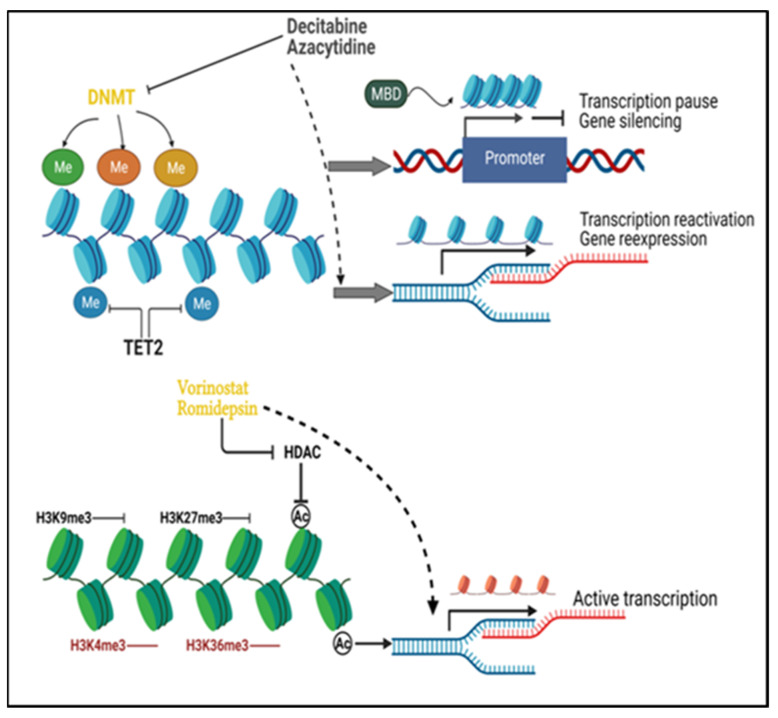
Principles of cancer epigenetic modifications and their drug targets. DNA methyltransferases (DNMTs) add methyl groups to DNA and maintain methylated DNA, while Tet methylcytosine dioxygenase 2 (TET2) removes the methyl groups from DNA. DNA methylation at the gene promoter impairs local binding of transcription factors and blocks transcription. Recruitment of methyl CpG binding domain (MBD) protein by the methylated DNA facilitates the heterochromatin formation and results in transcription repression. DNMT inhibitors such as decitabine and azacytidine will incorporate into the genome and degrade the activity of DNMT, reverse the aberrant DNA hypermethylation, and enable the re-expression of silenced genes. H3K9me3 and H3K27me3 serve as repressive histone marks, while H3K4me3 and H3K36me3 are active marks. Histone deacetylation is among the major repressive mechanisms of histone modification. Histone deacetylases (HDAC) inhibitors (e.g., vorinostat and romidepsin) inhibit histone deacetylation caused by HDAC to maintain active chromatin status for transcription. Me: DNA methylation; Ac: histone acetylation.

Inhibition of the histone methyltransferase G9a has been shown to be a potential target for the treatment of several malignancies [77,78,79]. Knockdown of G9a inhibited tumor growth and progression in mouse models of pancreatic carcinoma [80]. G9a expression was enriched in tumor-initiating cells (TICs) of non-small cell lung cancer (NSCLC), and loss of G9a led to reduced proliferation and sphere-forming capacity of TICs. In addition, the hypomethylation of select genes in response to G9a deprivation associates with favorable clinical prognosis in NSCLC patients [77]. Oncogenic driver MYC cooperates with G9a to epigenetically silence gene expression. Depletion of G9a abrogates the binding of MYC to chromatin, reverses the repression of MYC-suppressed genes, and hinders tumor growth [81]. Some preliminary studies have shown promising results suggesting dual inhibition of DNMT and G9a may be a therapeutic strategy in certain cancer models. Co-inhibition of DNMT and G9a showed high potential to inhibit cellular proliferation, to promote INF-stimulating genes and prolong the survival of tumor models in hematological lesions [82]. The expression of DNMT1 and G9a was shown to correlate with poor prognosis in HCC and dual inhibition of these two targets impaired cell growth in vitro and in vivo [83]. Similarly, G9a was associated with poor prognosis in bladder cancer and exhibited resistance to anti-PD1 therapy. The combination of dual inhibition of G9a and DNMT1 together with PD-L1 showed promising results, especially in inducing immunogenic cell death and adjusting the endogenous antitumor immune response [84]. Epigenetic drugs (Figure 1) are expected to have versatile potential, especially in preventing cancer recurrence and resistance, as well as in sensitizing to therapeutics during long-term treatments [5].

### 4.2. Epigenetic Agents

5-Aza-2-deoxycytidine (decitabine) and 5-azacytosine (azacytidine) are two classical DNMTi, both of which can reverse DNA hypermethylation by covalently trapping the DNMTs to DNA and leading to their degradation [3]. In the cells, these agents are converted to the triphosphate form and become physiologically active. Decitabine is incorporated into DNA while azacytidine binds mostly to RNA, but a small percentage of the converted product is incorporated into DNA as well [59]. Of note, decitabine possesses a half-life of only 12–25 min in patients, due to degradation by cytidine deaminase in the liver after the drug enters the bloodstream [85]. Decitabine has shown clinical benefit in hematological malignancies [86] and showed potential to sensitize to therapeutic response in solid tumors, for example, by improving chemosensitivity in refractory ovarian cancer patients [87].

Histone acetylation and deacetylation are catalyzed by histone acetyltransferases (HATs) and histone deacetyltransferases (HDACs). In addition to histones, HDACs can also bind to and catalyze non-histone proteins; the binding partners include p53 and transcription factors such as STAT, GATA1-3 [88]. HDAC inhibitors (HDACi) represent a group of epi-drugs that are extensively studied. They are categorized into three classes: hydroxamates (vorinostat, belinostat, panobinostat), benzamides (entinostat, chidamide), cyclic peptides (romidepsin), and aliphatic acids. Some of these compounds are being tested in clinical trials either alone or in combination with other anticancer drugs in various malignancies, from multiple myeloma and myelodysplastic syndrome to glioblastoma, ovarian cancer, and some other epithelial/solid tumors [89]. Several HDACi have been approved by the Food and Drug Administration (FDA) for the treatment of cutaneous T-cell lymphoma, including vorinostat, romidepsin, and belinostat [90].

Although vorinostat has not been shown to be as effective as single agent therapy in solid tumors in clinical trials, it has been proposed that this epi-agent be combined with other chemotherapy drugs to optimize therapeutic benefit. Most importantly, downregulation of oncogenes and upregulation of tumor suppressors is considered to be the main mechanism of action of vorinostat [90,91]. Interestingly, depsipeptide (romidepsin) not only caused histone deacetylation but also strongly demethylated the promoter of some genes, including *p16*, *SALL3*, and *GATA4*. Moreover, attenuated binding of DNMT1 together with decreased expression of H3K9 methyltransferases G9a and SUV39H1 was suggested to underlie the indirect demethylating activity of depsipeptide [92].

## 5. Transposable Elements

**Figure 2:**
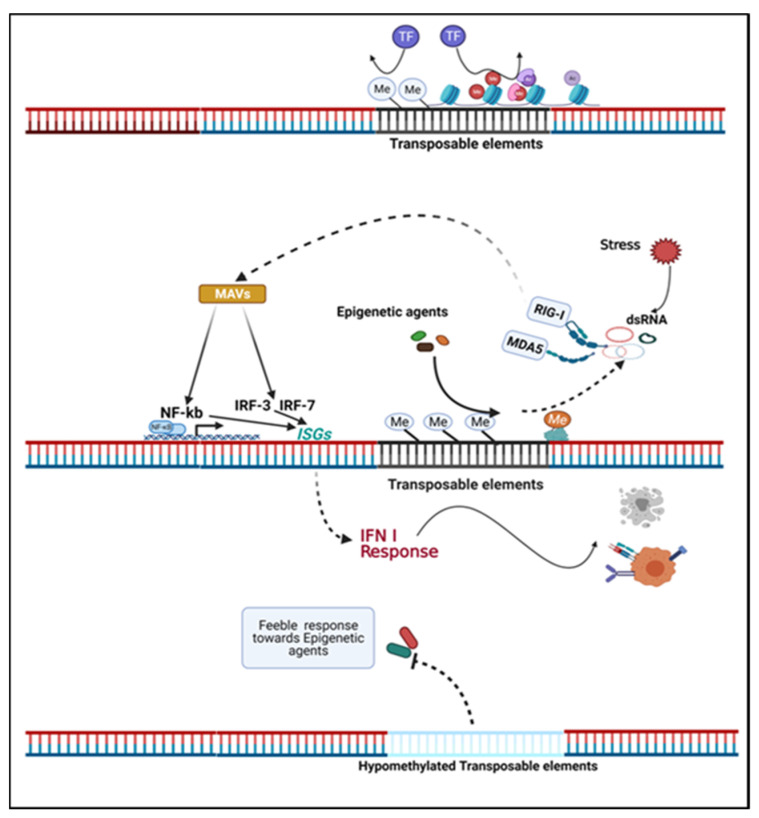
Transposable elements determine the inherent immunogenicity and response of tumor cells to epigenetic agents. Transposable elements (TE) in the genome are typically not actively transcribed but can be stimulated by stress and epigenetic agents. Endogenous retroviruses (ERVs) compose a major part of TE. Regionally hypermethylated ERVs are transcriptionally inactive, and repressive histone modifications at ERVs loci disturb the access of genome for transcription factors (TF). Epigenetic agents potentiate the transcription of ERVs into nucleic acids that mimic a virus infection. The transcription product of ERVs, dsRNA, are sensed by cytosolic sensors: retinoic acid-inducible gene I (RIG-I) or melanoma differentiation-associated gene 5 (MDA5). The resulting signal is transduced by mitochondrial antiviral proteins (MAVs) and leads to NF-kb and interferon regulated factors (IRF) translocation into the nucleus, inducing the expression of interferon-stimulated genes (ISGs) and type I IFN response and results in tumor cell apoptosis or enhanced expression of tumor associated antigens. Hypomethylated ERVs may be a characteristic epigenetic feature in tumor cells and may perturb cellular responses to epigenetic agents. Inherent ERV patterns and regional epigenetic modifications may provide predictive value for epigenetic therapy [52,100,101].

Endogenous retroviruses (ERVs), as a subset of TEs, may account for up to 8% of the human genome. TEs were once interpreted as “genetic parasites” because of their non-coding roles. However, it was later found that these elements can be actively transcribed into nucleic acids or proteins that resemble pathogen-associated molecular patterns (PAMPs) and are recognized by pathogen recognition receptor, resulting in an immune response that resembles an antiviral response [7,93]. The TE can be divided into two classes: 1. Class I, also known as retrotransposons, contains long terminal repeats (LTR)/ERV, long and short interspersed nuclear elements (LINEs and SINEs); 2. Class II, the main component is DNA transposons [7]. Retrotransposons are classified according to an alternative classification into either autonomous or non-autonomous elements. The former contains long terminal repeats (LTR) and non-LTR retrotransposons—also referred to as LINEs and the latter contains SINEs [94]. The ability of ERV to elicit an antiviral immune response (Figure 2) can be explained by the fact that nucleic acids produced by viral infections or endogenous retroelements are normally distinct from host cellular RNA and are therefore recognized as PAMP. Retinoic acid-inducible gene I (RIG-I) and Toll-like receptors (TLR) are two important RNA sensors. RIG-I recognizes cytosolic viral RNA, while TLR recognizes extracellular viral RNA endocytosed in endolysosomes [95]. Indeed, dsRNA is recognized by TLR-3, ssRNA is recognized by TLR-7 and TLR-8, and foreign DNA is recognized by TLR-9. Melanoma differentiation-associated gene 5 (MDA5) also serves as a sensor for intracellular dsRNA [96]. DNA demethylating agents can restore the expression of ERVs in tumor cells, placing the cells in a mock virus-infected state that then impairs the cell growth and proliferation [97,98]. Viral or endogenous RNA sensing leads to downstream activation of NF-kB and interferon-regulated factors, coupled with an IFN type I response and activation of a number of interferon stimulated genes (ISGs) [99,100]. Type I and III IFN responses activate transcription of ISGs through JAK/STAT pathways, and type II IFN (IFN-γ) response transduces signaling through STAT1 phosphorylation and nuclear translocation and subsequent binding to the promoters of IFN-γ induced genes [101].

A recent analysis from The Cancer Genome Atlas (TCGA) demonstrated the predictive value of human ERVs (hERVs) present in clear cell renal carcinoma cells in response to anti-PD1 therapy and showed that variable signatures of hERVs correlated with differential survival: the RIG-I like up (up 50th percentiles) implied longer overall survival compared to the RIG-I like down (down 50th percentiles) group [102]. Interestingly, only a basal level of genes within the antiviral pathway was shown in the DNMT1 hypomorph cells and the upregulation of antiviral related genes can be achieved by DNMT1 depletion [74]. High expression of ERVs in tumors positively correlated with an efficient antiviral response [103]. Recently, an interesting discovery was made regarding how epigenetic modifications and agents can alter the transcription of TEs. This study showed that resistance of triple-negative breast cancer cells to taxanes can be attributed to loci enriched in hypomethylated TEs but abundant in H3K27me3, which negatively affected TE transcription and viral mimicry, thereby attenuating intracellular antiviral immunity and enhancing the sustainability of taxane-resistant cells [52].

Endogenous dsRNA, which is triggered and reactivated by stimuli, represents an important element of the antiviral immune response. Apart from viral infections, dsRNA may arise from tissue stress, damage, and necrosis [93,104]. It has been suggested that dsRNA shares the same signaling pathway as dsDNA, which can be activated by viral DNA infection [99]. Notably, recognition of viral RNA leads not only to apoptosis but also to pyroptosis, a state of inflammasome-mediated cell death accompanied by disrupted cell membrane integrity and release of cytoplasmic content from cells [105]. A recent finding reported that the protein expression of *AGO1x* interfered with dsRNA accumulation in breast cancer cells and hampered the dsRNA-induced interferon response, leading to refractory cell growth. Loss of *AGO1x* expression restored the dsRNA and interferon response and eventually led to more apoptosis [106].

Epi-agents induce retroelements sensed by RIG-I and MDA5 which affect the intracellular glucose hydrolysis, resulting in energy depletion and tumor cell death. In addition, this effect is coupled with altered mitochondrial metabolism to compensate for ATP, and tumor cell death (necroptosis) is independent of caspase-mediated apoptosis but closely associated with BCL2 [107].

## 6. Epigenetic Targeting Meets Immune Check Point Inhibition: Does the Union Empower?

### 6.1. Tumor-Infiltrating Immune Cells in Gliomas

The tumor microenvironment of gliomas is unique in part due to the blood–brain barrier (BBB). Of note, glioblastoma is referred to as an immunogenic “cold tumor” because of the lack of tumor antigen expression, the absence of antigen presentation to T cells, and the high level of immune checkpoints on infiltrating lymphocytes [108]. Indeed, lymphocytes require adhesion signals on endothelial cells to migrate into the brain, additionally, naive T cells are not normally present in the central nervous system while T cells that penetrate the BBB are patrolling T cells and regulatory T cells that prevent inappropriate inflammatory responses [109].

A relevant analysis of TCGA data revealed that monocytes, activated NK cells, macrophages, and eosinophils among other infiltrative immune cells, correlated with survival of glioblastoma patients, with the abundance of macrophages indicating poorer survival, while the others were associated with better survival [110].

A retrospective study of immunohistochemical analysis of tissue samples from 43 glioblastoma patients concluded that among the infiltrating immune cells, lymphocytes were sparsely distributed compared to macrophages, but a lower amount of CD4/CD8 infiltrating lymphocytes (TILs) was associated with better survival [111].

Genetic alterations in tumors also correlate with TILs in the tumor microenvironment. For instance, TILs are enriched in *NF1* and *RB1* mutated gliomas but depleted in *EGFR*-amplified and *PTEN*-deleted gliomas. Interestingly, *IDH*-wildtype glioma is usually associated with more lymphocyte infiltration and PD-L1 expression while *IDH*-mutant gliomas have less IFN-γ and lower infiltration of CD8^+^ and CD4^+^ T cells [112]. Methylation chip-based analysis of gliomas found that there was no dramatic difference in the extent of immune cell infiltration between long-term and short-term survivors [113]. A study of 519 glioblastoma patients indicated that long-term survivors were more likely to have extensive T cell infiltration than short-term survivors, with high CD8^+^ infiltrating T cells indicating long-term survival [114]. A recent study uncovered the correlation between infiltrated T cells and overall survival in glioma patients. Patients with T cell-deficient gliomas presented a longer survival than the T cell-enriched group; nevertheless, CD8^+^ T cell-dominant group predicted a better survival as compared with the CD4^+^ T cell-dominant group. Notably, fewer infiltrated macrophages were found in the *IDH*-mutated gliomas [115].

*IDH*-mutated tumors were found to express less IFN-γ inducible chemokines such as CXCL10, which was further confirmed by the introduction of *IDH1* mutation which decreased CXCL10 expression and reduced the number of T cells in a glioma mouse model. Furthermore, mutant IDH1 inhibitor led to increased survival in preclinical glioma models and led to increased CXCL10 expression and TILs [116]. Similarly, Weenink et al. quantified the TILs in both lower and high-grade glioma (LGG and HGG) samples and discovered that LGG contained fewer CD8^+^ T cells, which was related to the lower expression of CXCL9, CXCL10, and ICAM1, the relative absence of TILs in LGG was thought to potentially affect the therapeutic efficacy of immune checkpoint inhibitors in this context [117].

### 6.2. Combination of Epigenetic Drugs with Immune Checkpoint Inhibitors

The epigenetic modifications inherent to the tumor may reflect its immunogenic properties in the antitumor microenvironment. Epigenetic agents have been shown to restore the vulnerability of tumor tissues to therapeutic modalities. For example, treatment of colon and ovarian cancer cell lines with DNMTi enhanced antigen presentation and cancer testis antigens at the transcriptional and translational levels [118]. In non-small cell lung cancer, analysis of CpG-methylation assays and bisulfite sequencing revealed that *CTLA-4* and *PD1* methylation levels were reduced compared to normal tissues and epigenetic changes were inversely correlated with gene expression [119]. Encouraging results showed that epigenetic agents enhance antitumor immunity, especially when combined with certain compounds, as demonstrated in a number of reports. In a study with decitabine on glioblastoma cells and patient samples, it was found that tumor cells showed increased expression of MHC I and tumor-associated antigens after decitabine treatment, and T cells presented an upregulated Fas ligand (CD95) in association with increased levels of INF-γ, TNF-α, IL-5, and CD107A (functional parameters of degranulation of cytotoxic T cells via the Fas pathway) of NY-ESO-1 specific T cells and concluded that the epigenetic agent sensitizes glioblastoma to the functionality of specific T cells [120].

Treatment of lung cancer cells with azacytidine was shown to alter a variety of immune-related gene expression, including upregulation of HLA and IFN-γ and its downstream signaling factors. Azacytidine led to increased PD-L1 and CD80/CD86 (CTLA-ligands), providing a rationale for combining of azacytidine with immune checkpoint blockade to overcome immune evasion of tumor cells [121]. Ishibashi et al. showed the inverse correlation of HLA-G expression with prognosis in breast cancer patients. Decitabine treatment increased HLA-G expression in tumor cells and enhanced recognition of these cells by specific CD4^+^ helper T lymphocytes, suggesting a combination of decitabine with HLA-G targeting to improve T cell-based immunotherapy [122]. Similar efficacy was observed in mouse GL261 glioma cells. Decitabine potentiated the immunogenic signature in glioma-initiating GL261 cells by increasing the expression of FasL and MHCI which enhanced tumor recognition and killing by CTLs [123]. The new generation DNMTi guadecitabine has shown the potential to alter the antitumor microenvironment by increasing MHCI expression and enhancing IFN-γ response in breast cancer cells. Moreover, tumor growth was significantly slowed when guadecitabine was used together with anti-PD-L1 therapy in a mouse model [124]. Similarly, azacytidine enriched effective immune cells via type I IFN signaling. Moreover, the triple combination of azacytidine, HDACi, and PD-1 antibody showed the greatest antitumor potency in a mouse ovarian tumor model [125]. Combinatorial use of HDACi (SAHA and CI994) with the PD-1 inhibitor showed promising efficacy in the mouse model of urothelial bladder cancer. HDACi was shown to facilitate delayed immune recognition by upregulating the expression of associated genes such as *NGK2D* and *HSP70*. Meanwhile, it has been suggested that fully activated T cells are not sufficient for intact antitumor immunity, but that pre-exposure of tumor cells to agents such as HDACs will optimize the antitumor immunity [126]. As such, HDACi CG-745 modulated the immune microenvironment by increasing the proportion of cytotoxic T cells and NK cells and decreasing the suppressive immune components such as regulatory T cells and myeloid-derived suppressor cells and favored the anti-PD1 therapy in a synergistic fashion [127].

The elementary factors that determine the response of cancer cells to immune checkpoint blockade include the tumor mutational burden, immune phenotype of tumor microenvironment, and immune escape of tumors [71]. The inherent and acquired epigenetic modifications within the loci of immune checkpoint genes may contribute to resistance to immune checkpoint inhibitors as only a subset of patients respond to immunotherapy. The relevant epigenetic modifications may be potent predictive biomarkers for immune checkpoint therapy and can be targets in a combination strategy to increase therapeutic benefit. Increased expression of PD-L1 upon azacytidine was shown to elevate the response to anti-PD1 therapy [128]; similarly, high levels of PD-L1 and TIL were associated with positive response to anti-PD1/PD-L1 therapy [129]. Similarly, melanoma patients with low PD-L1 expression and low TIL count did not respond to anti-PD1 therapy. Notably, abundant miRNA negatively regulated PD-L1 expression across multiple cancers and contributed to resistance to immune checkpoint inhibitors [130]. Lower *CTLA-4* methylation in melanoma samples indicated a better response to anti-PD1 or anti-CTLA-4 therapy [131] and increased level of *PD-1*, *CTLA-4*, or *PD-L1* was found to correlate with DNA hypomethylation across many types of tumors such as non-small cell lung cancer, lower grade gliomas (LGG), and head and neck squamous cell carcinoma [132].

Several clinical trials on the combinatorial approach of epigenetic agents and immune checkpoint inhibitors for various tumors are still ongoing, and their therapeutic effects and potential side effects are being monitored (Table 1). As a novel concept that showed exciting results in several preclinical and clinical studies, the combination of immune-checkpoint inhibitors with epigenetic agents may provide increased therapeutic benefit. Hopefully, these studies will add to our current knowledge of the clinical utility and limitations of epigenetic agents and combinatorial strategies for the benefit of patients.

## 7. Conclusions

Cancer-associated epigenetic modifications play a central role in suggesting an appropriate therapeutic strategy. Previous studies have shown that epigenetic agents, in addition to being an “epigenetic editor”, also can activate silenced tumor suppressor genes and cellular antiviral signaling pathways, and tumor-associated antigens and immune-checkpoints. The epigenetic landscape of tumors and its influence on tumor phenotype, microenvironment, and the interaction between epigenetics and immune plasticity with respect to tumorigenesis and progression are of great scientific interest. Given the complexity and diversity of epigenetic modifications in different tissues, tumor grades, and therapy-related potential alterations, more comprehensive knowledge is needed to appropriately design preclinical studies and clinical trials accompanied by interdisciplinary expertise.

## Figures and Tables

**Table 1:** Clinical trials of epigenetic agents combined with immune checkpoint inhibitors for cancer therapy.

Identifier	Malignant Conditions	Therapeutics (Single or Combined)	Start Date	Results
NCT02608268	Advanced solid tumors	1. MBG453 (Tim3 antibody) 2. PDR001 (PD-1 antibody) 3. Decitabine	November 2015	Recruiting
NCT03066648	Acute myeloid leukemia or high risk myelodysplastic syndrome	1. Decitabine/Azacytidine 2. PDR001 3. MBG453	July 2017	Recruiting
NCT03019003	Head and neck cancer	1. ASTX 727 (oral decitabine) 2. Durvalumab (PD-L1 antibody)	March 2017	Recruiting
NCT03161223	Relapsed or refractory peripheral T-cell lymphomas (PTCL)	1. Durvalumab (PD-L1 inhibitor) 2. Romidepsin 3. 5-azacytidine 4. Pralatrexate	May 2018	Recruiting
NCT01928576	Non-small cell lung cancer (NSCLC)	1. Azacytidine 2. Entinostat 3. Nivolumab	August 2013	Recruiting
NCT04611711	PD-1 monoclonal antibody-resistant digestive system tumors	1. Decitabine 2. TQB2450 (PD-1 inhibitor) 3. Anlotinib (VEGFR inhibitor)	November 2020	Recruiting
NCT02890329	Relapsed or refractory myelodysplastic syndrome or acute myeloid leukemia	1. Decitabine 2. Ipilimumab (CTLA-4 antibody)	September 2016	Recruiting
NCT04277442	Newly diagnosed TP53 mutated acute myeloid leukemia	1. Decitabine 2. Nivolumab (PD-1 inhibitor) 3. Venetoclax (Bcl-2 inhibitor)	February 2020	Recruiting
NCT02397720	Refractory/relapsed or newly diagnosed acute myeloid leukemia	1. Azacytidine 2. Ipilimumab 3. Nivolumab	April 2015	Recruiting
NCT02816021	Metastatic melanoma	1. Azacytidine 2. Pembrolizumab (PD-1 inhibitor)	February 2017	Recruiting
NCT03233724	Inoperable locally advanced or metastatic NSCLC, and esophageal carcinomas, or pleural mesotheliomas	1. Oral decitabine 2. Tetrahydrouridine (inhibitor of cytidine deaminase) 3. Pembrolizumab (PD-1 inhibitor)	April 2018	Recruiting
NCT02959437	Advanced solid tumors and previously treated stage IIIB or stage IV non-small cell lung cancer and stage IV microsatellite-stable colorectal cancer	1. Azacytidine 2. Pembrolizumab 3. Epacadostat (indoleamine2,3-dioxygenase inhibitor) 4. INCB057643 (BET inhibitor) 5. INCB059872 (LSD1 inhibitor)	February 2017	Recruiting
NCT02546986	Locally advanced or metastatic non-small cell lung cancer	1. CC-486 (oral azacytidine) 2. Pembrolizumab	October 2015	Recruiting
NCT04250246	Melanoma and NSCLC resistant to anti-PD1/PDL1	1. Ipilimumab + Nivolumab + Guadecitabine 2. Ipilimumab + Nivolumab	March 2020	Recruiting
NCT03765229	Melanoma	1. Entinostat 2. Pembrolizumab	March 2019	Recruiting
NCT02437136	NSCLC, melanoma and mismatch repair-proficient colorectal cancer	1. Entinostat 2. Pembrolizumab	July 2015	Recruiting
NCT03024437	Advanced renal cell carcinoma	1. Atezolizumab (PD-L1 inhibitor) 2. Bevacizumab (VEGF inhibitor) 3. Entinostat	May 2017	Recruiting
NCT04708470	Solid tumors, metastatic checkpoint refractory HPV-associated tumors, microsatellite stable colon cancer	1. Bintrafusp Alfa (bifunctional fusion protein composed of the extracellular domain of the TGF-β receptor II fused to an IgG1 antibody blocking PD-L1) 2. NHS-IL12 3. Entinostat	August 2021	Recruiting
NCT02915523	Advanced epithelial ovarian cancer	1. Entinostat 2. Avelumab (PD-L1 inhibitor)	January 2017	Recruiting
NCT03250273	Previously treated unresectable/metastatic cholangiocarcinoma and pancreatic cancer	1. Entinostat 2. Nivolumab	November 2017	Recruiting
NCT03854474	Locally advanced and metastatic urothelial carcinoma	1. Pembrolizumab 2. Tazemetostat (EZH2 inhibitor)	May 2019	Recruiting
NCT02453620	Unresectable or locally advanced or metastatic Her2-negative breast cancer	1. Entinostat 2. Ipilimumab 3. Nivolumab	November 2015	Recruiting
NCT02395627	Hormone receptor expressing advanced breast cancer	1. Vorinostat 2. Tamoxifen 3. Pembrolizumab	May 2015	Recruiting

## Data Availability

Not applicable.
